# Free-breathing 3D phase-sensitive inversion recovery late gadolinium enhancement at 3.0 Tesla: reliability and image quality in ischemic and non-ischemic cardiomyopathy in comparison with multiple breath-hold 3D imaging

**DOI:** 10.1186/1532-429X-17-S1-P97

**Published:** 2015-02-03

**Authors:** Maurice Bizino, Jacob Amersfoort, Qian Tao, Rob J van der Geest, Hildo J Lamb

**Affiliations:** LUMC, Leiden, Netherlands

## Background

In both ischemic (ICM) and non-ischemic (NICM) cardiomyopathy late gadolinium enhancement (LGE) is an important cardiovascular magnetic resonance (CMR) technique. LGE CMR, traditionally performed in 2D during multiple breath-holds (MB), is challenging for vulnerable patients and subject to slice misregistration. Therefore, LGE CMR during free-breathing (FB) is more robust and enables to perform 3D acquisition, increase resolution and apply phase-sensitive inversion recovery (PSIR). We developed and clinically tested a high spatial resolution PSIR LGE sequence using a respiratory navigator in both ICM and NICM patients. Reliability and image quality were compared to a multiple breath-hold 3D approach (MB-3D).

## Methods

Approval was waived by the institutional ethical committee. Between May 2012 and November 2013 FB-3D and MB-3D sequences were acquired in patients suspected of ICM and NICM. Data of 48 patients (36 men; mean age ± standard deviation: 60.8 years ± 10.9; 34 ICM) were retrospectively collected. LGE sequences were acquired using a 3.0 Tesla MR system. For both FB-3D (1.68 x 1.68 x 3.4 mm) and MB-3D (1.86 x 2.8 x 10 mm) a PSIR sequence was used. Multiplanar reformats with high resolution (FB-3D-HR; 0.91 mm isotropic voxels) and low resolution (FB-3D-LR; 1.86 x 2.8 x 10 mm) were constructed. LGE mass and image quality (SNR, CNR and edge sharpness) were compared using the Friedman test, Wilcoxon signed rank test, spearman correlation and Bland-Altman analysis.

## Results

**Figure 1:**
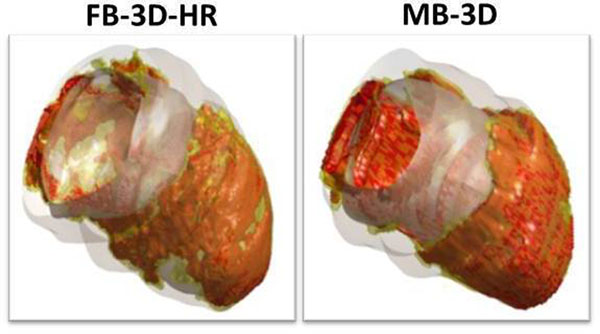
**42-year old female patient a previous anterior myocardial infarction admitted under the suspicion of a sub-acute inferolateral infarction.** CMR revealed large areas of LGE anterior and inferolateral. Also, a true apical thrombus was detected. 3D reconstructions of the entire left ventricle are dysplayed. Note the increased spatial resolution in the FB-3D-HR images with higher edge sharpness, apparent in for example areas with microvascular obstruction (anterior and lateral wall).

**Figure 2:**
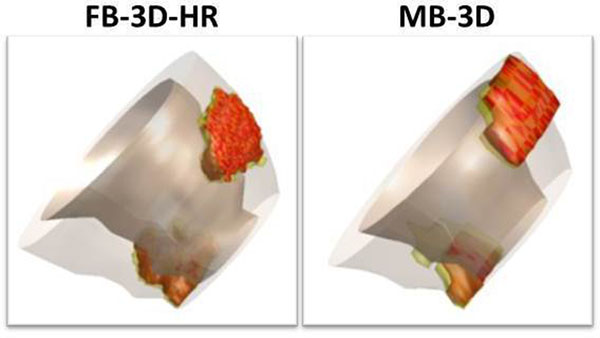
**49-year old male patient admitted with thoracic pain related to breathing with elevated serum creatine kinase and troponine values.** CMR reveals LGE in the epicardial apico-anterior wall and extending into the pericardium, and in the epicardial apico-inferior wall suggesting perimyocarditis. Appreciate the high contrast in the FB-3D images and sharp delineation of LGE areas in the FB-3D-HR 3D reconstruction.

34 ICM patients (23 chronic MI; 4 sub-acute MI; 7 acute MI) and 14 NICM were included (5 dilated cardiomyopathy; 4 hypertrophic cardiomyopathy; 3 peri-/myocarditis; 2 other). In figures 1 and 2 3D reconstructions of two patients are shown.In the complete dataset of 48 patients, there were no significant differences between FB-3D-HR, FB-3D-LR and MB-3D datasets in terms of LGE mass (FB-3D-HR: (median [interquartile range]): 8.5 g [4.4-19.9]; FB-3D-LR: 10.7 g [4.5-22.9]; MB-3D: 9.8 g [5.1-19.8]; p = 0.099). LGE mass correlated well (FB-3D-HR vs MB-3D: r=0.922, p=0.01; FB-3D-LR vs MB3D: r=0.922, p=0.01) with Bland Altman analysis indicating good agreement for FB-3D-HR vs MB-3D (mean difference ± SD: 1.15 ± 5.45 gram) and FB-3D-LR vs MB-3D (mean difference ± SD: 0.00 ± 5.47 gram). SNR was higher in both FB-3D-HR (median [interquartile range]: 244 [192-326], p=0.001) and FB-3D-LR (825 [498-1417], P<0.001) as compared to MB-3D (200 [146-263]). CNR was also higher in both FB-3D-HR (226 [164-277], p<0.001) and FB-3D-LR (691 [480-1086], p<0.001) as compared to MB-3D (216 [162-260]). Edge sharpness was 25% higher in FB-3D-HR as compared to MB-3D (0.21 [0.19-0.23] vs 0.17 [0.15-0.20], p<0.001).

## Conclusions

Free-breathing 3D phase-sensitive inversion recovery LGE CMR enables reliable myocardial scar tissue assessment with significantly improved image quality as compared with multiple breath-hold 3D imaging in ischemic and non-ischemic cardiomyopathy.

## Funding

No funding.

